# ﻿A new species of the rib-degenerated loach, genus *Protocobitis* (Cypriniformes, Cobitidae), from Guangxi, China

**DOI:** 10.3897/zookeys.1228.131341

**Published:** 2025-02-21

**Authors:** Zhi-Xian Qin, Ye-Wei Liu, Si-Yu Zhang, Jing-Song Shi, Li-Na Du, Jia-Jun Zhou

**Affiliations:** 1 Key Laboratory of Ecology of Rare and Endangered Species and Environmental Protection (Guangxi Normal University), Ministry of Education, Guilin, Guangxi 541004, China; 2 Guangxi Key Laboratory of Rare and Endangered Animal Ecology, College of Life Science, Guangxi Normal University, Guilin, Guangxi 541004, China; 3 Guangxi Key Laboratory for Forest Ecology and Conservation, College of Forestry, Guangxi University, Nanning, Guangxi 530004, China; 4 Institute of Zoology, Chinese Academy of Sciences, Beijing 100101, China; 5 Institute of Vertebrate Paleontology and Paleoanthropology, Chinese Academy of Sciences, Beijng 100044, China; 6 Zhejiang Forest Resource Monitoring Center, Hangzhou, Zhejiang 310020, China; 7 Zhejiang Forestry Survey Planning and Design Company Limited, Hangzhou 310020, China

**Keywords:** Cavefish, mitochondrial gene, Pearl River, taxonomy

## Abstract

A new species of the genus *Protocobitis* is described based on morphological comparisons and molecular analyses from specimens of a subterranean tributary of the Hongshui River, Lingyun County, Baise City, and a cave in Jinya Township, Fengshan County, Hechi City, Guangxi, China. Both morphological and molecular data support the validity of *Protocobitislongibarba***sp. nov.** The new species can be distinguished from congeners by the following combination of characteristics: whole body except for head and area between pectoral-fin and pelvic-fin origin sparsely covered with minute scales; barbels elongate; five or six branched pectoral-fin rays and four branched pelvic-fin rays; vertebrae 4+42. Maximum-likelihood and Bayesian-inference phylogenetic trees exhibited congruent topological structures, exhibiting high node support for the monophyly of *Protocobitislongibarba* (BPP = 1; BS = 100), which was clustered with the other congeners.

## ﻿Introduction

The unique karst landforms of the Guangxi Zhuang Autonomous Region (hereinafter referred to as Guangxi) have resulted in extensive surface water and ground water systems, providing ideal conditions for the evolution and adaptative radiation of cavefish species. The perpetual absence of light in caves prevents photosynthesis, leading to a limited food supply primarily sourced from surface water exchange. This scarcity of food presents considerable challenges in providing adequate nutrition for fish reproduction. Consequently, populations of karst cavefish, such as *Sinocyclocheilushyalinus* Chen & Yang, 1993, are extremely rare (Chen et al. 1994; Jeffery 2001, 2009; Zhao and Zhang 2009; Liang et al. 2011; Fan et al. 2024).

The genus *Protocobitis* Yang, Chen & Lan, 1994 was initially described based on specimens collected from Du’an County, Guangxi, with the type species *Protocobitistyphlops* Yang, Chen & Lan, 1994 (Yang et al. 1994). Endemic to Guangxi, this genus is a typical cave-dwelling fish species, displaying distinctive characteristics such as the absence of eyes, pigment degeneration leading to transparency or semitransparency, elongate barbels, reduction or absence of body scales, and tiny cranial bones (Zhao and Zhang 2006); thus, it demonstrates a high degree of adaptation to cave life. Four valid species have been recognized within the genus, including *P.anteroventris* Lan, 2013 and *P.longicostatus* Zhou, Qin, Du &Wu, 2024 from Baise City, *P.typhlops* from Hechi City, and *P.polylepis* Zhu, Lv, Yang & Zhang, 2008 from Nanning City (Yang et al. 1994; Zhu et al. 2008; Lan et al. 2013; Zhou et al. 2024). All known *Protocobitis* species are eyeless and exhibit varying degrees of rib degeneration.

Five specimens of *Protocobitis* were collected in February 2024 from a subterranean tributary of the Hongshui River in Luolou Town, Lingyun County, Baise City, and two collected in May 2024 from a cave in Jinya Township, Fengshan County, Hechi City, Guangxi, China. Results of our morphological and molecular analyses indicate that these loach specimens represent a new species of *Protocobitis*, which is described herein.

## ﻿Materials and methods

All field collections abided by the rules of the Fisheries Law of the People’s Republic of China. All activities conformed to the Laboratory Animal Guidelines for the Ethical Review of Animal Welfare (GB/T 35892-2018). After euthanizing the collected fish specimens with excessive anesthetic clove oil, the right pelvic fins were excised and placed in 95% alcohol for subsequent DNA sequencing, then the whole-body specimens fixed in 10% formalin and transferred to 75% ethanol for morphological study. Specimens were preserved at the Kunming Natural History Museum of Zoology, Kunming Institute of Zoology (**KIZ**), Chinese Academy of Sciences (CAS), and Zhejiang Forest Resource Monitorign Center (ZJFRF), Hangzhou, Zhejiang. Counts and measurements followed Yang et al. (1994). All measurements were taken point-to-point with dial calipers to the nearest 0.1 mm. X-ray scanning and three-dimensional (3D) reconstructions were conducted using nano-computerized tomography (CT) with a GE V|tome|X m dual tube 300/180 kV system at the Key Laboratory of Vertebrate Evolution and Human Origins, Institute of Vertebrate Paleontology and Paleoanthropology (IVPP), CAS. The specimens were scanned with an energy beam of 80 kV and a flux of 80 × µA using 360° rotation, then reconstructed into a 4 096 × 4 096 matrix of 1 536 slices. The final CT reconstructed skull images were exported with a minimum resolution of 8.9 μm. Skull images were exported from the virtual 3D model reconstruction using Volume Graphics Studio v. 3.4.0.

The extraction, amplification, and sequencing of genomic DNA were conducted by Tsingke Biotechnology Co., Ltd (China). Partial sequences of the mitochondrial *cytochrome c oxidase subunit I* (COI) and cytochrome *b* (cyt *b*) were sequenced and submitted to GenBank (accession: PP866712–PP866715 for COI, and PP868402–PP868405 for cyt *b*). The sequencing results were manually checked, corrected, and assembled using SeqMan within the Lasergene v. 7.1.0 package, DNASTAR, Inc., Madison Wis. The assembled sequences were aligned using MEGA v. 7.0 (Kumar et al. 2016) for multiple comparisons, and redundant segments were trimmed to obtain consistent sequences for further analysis. Genetic diversity analyses and haplotype filtering were performed using DnaSP v. 5 (Librado and Rozas 2009).

The complete mitochondrial genomes of 14 cobitid species and two botiid species (*Parabotiafasciata* Dabry de Thiersant, 1872 and *Leptobotiaelongata* Bleeker, 1870) were retrieved from GenBank to serve as the outgroup. The phylogenetic placement of *Protocobitislongibarba* was determined using maximum likelihood (ML) and Bayesian inference (BI) implemented in the CIPRES Science Gateway (Miller et al. 2010). The ML tree was reconstructed using RAxML-HPC v. 8 (Stamatakis 2014), with 1,000 rapid bootstrapping iterations. The BI tree was constructed using MrBayes in XSEDE v. 3.2.7a (Ronquist et al. 2012). Two parallel runs were performed, with four Markov chains starting from a random tree. The chains were run for five million generations and sampled every 100 generations. The first 25% of sampled trees were discarded as burn-in, and the remaining trees were used to obtain a consensus tree and estimate Bayesian posterior probabilities (BPPs). The phylogenetic trees were viewed and edited using FigTree v. 1.4.4 (Rambaut 2009). Uncorrected pairwise distances (1000 replicates) based on concatenated dataset of mitochondrial COI and cyt *b* sequences was estimated using MEGA v. 7.0 (Kumar et al. 2016).

## ﻿Results

### ﻿ Protocobitis longibarba sp. nov.

Taxon classificationAnimaliaCypriniformesCobitidae

9E6B0E0B-D766-5E32-BE81-BA4AD665917E

https://zoobank.org/50F311B3-8458-426E-A76B-808847C94DB3

Fig. 1 , Table 1

#### Type material.

***Holotype*.** • KIZ2024000004, male, 44.0 mm standard length (SL), Yangcun Village, Luolou Town, Lingyun County, Baise City, Guangxi, China, from a subterranean tributary of the Hongshui River; 24.4392°N, 106.7409°E, collected by J.J. Zhou, Y.W. Liu & S.P. Zhou; 15 February 2024. ***Paratypes*.** • KIZ2024000001–3, female, 51.1–51.9 mm SL, KIZ2024000005, male, 44.0 mm SL, ZJFRF2402010, male, 53.5 mm SL; five specimens, collected with holotype • KIZ2024000006–7, male, 39.5–43.1 mm SL, two specimens, Liangfeng Cave, Shima Lake, Jinya Township, Fengshan County, Hechi City, Guangxi, China; 24.5587°N, 106.8655°E; collected by Y.W. Liu; 23 May 2024.

#### Diagnosis.

*Protocobitislongibarba* can be distinguished from all other species of *Protocobitis* by the following combination of characteristics: whole body, except for head and abdomen, sparsely covered with minute scales (vs scaleless in *P.anteroventris*, scales present along midline of body in *P.typhlops*; barbels elongate; 5–6 branched pectoral fin rays (vs seven in *P.anteroventris*, *P.longicostatus*, and *P.polylepis*); four branched pelvic-fin rays (vs five in other *Protocobitis* species); caudal-peduncle height 34.9%–58.6% of its length (vs 64.1%–65.7% in *P.polylepis*, 27.9%–43.3% in *P.anteroventris*); head width 7.3%–10.3% of SL (vs 5.4%–6.6% in *P.anteroventris*); head height 50.2%–80.6% of lateral head length (vs 45.7%–49.5% in *P.longicostatus*, 43.8%–46.8% in *P.anteroventris*).

#### Description.

**Figure 1.**
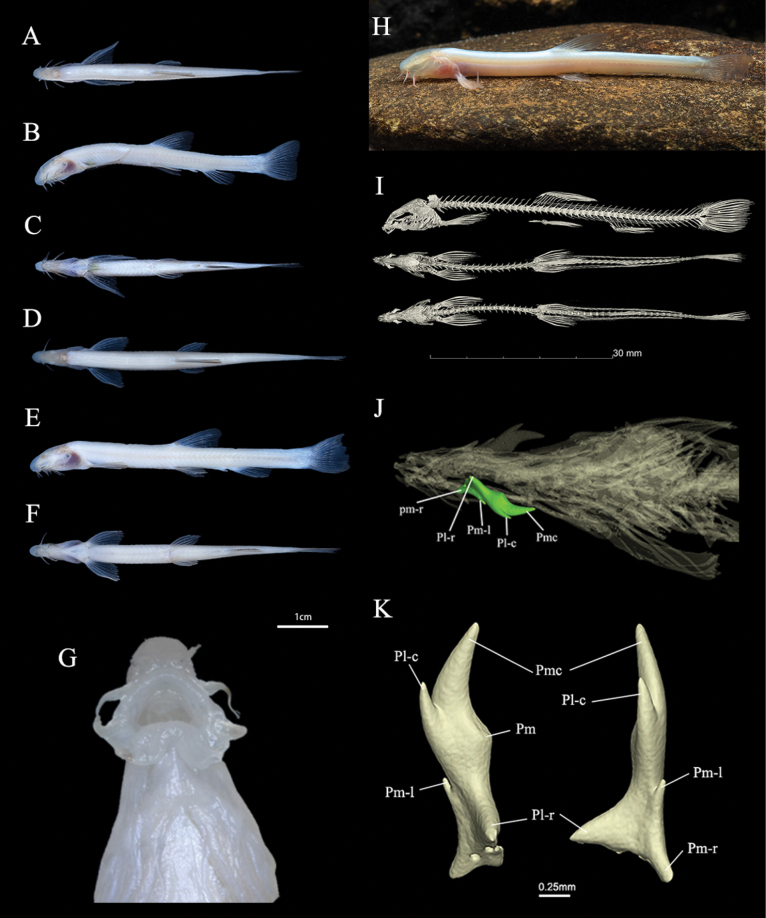
Morphometric characters of *Protocobitislongibarba* sp. nov. **A–C** lateral, dorsal and ventral views of male, holotype KIZ2024000004 **D–F** lateral, dorsal and ventral views of female, paratype KIZ2024000003 **G** ventral view of mouth **H** live male **I** lateral and ventral views of skeleton, paratype ZJFRF2402010 **J, K** suborbital spines (Abbreviations: Pmc, Processus mediocaudalis; Pl-c, Processus latero-caudalis; Pm, Processus medialis; Pm-l, Processus medio-lateralis; Pl-r, Processus latero-rostralis; Pm-r, Processus medio-rostralis).

Body elongate; maximum body width located immediately anterior to dorsal fin. Dorsal and ventral profiles almost straight except for slightly convex anus and base of fin. Snout obtuse. Head short, higher than width, roughly triangular in dorsal view. Nostrils closely set, nearer to snout tip than to the operculum, anterior nostril in short tube. Eyeless. Suborbital spine bifid, relatively thick and short, with strong mediolateral process in front of cavity of eye, length of laterocaudalis processus nearly half of mediocaudalis process, four strumae in base of mediorostralis process (Fig. 1J, K). Mouth inferior and arched, in vertical line of nostrils. Lips thin and smooth, each side of middle of lower lip with pair of developed fleshy mental lobes (Fig. 1G). Inner surface of mouth densely covered with numerous papillae, and outer edge of upper jaw neatly arranged with row of small nodules. Three pairs of barbels, inner rostral barbel reaching corner of mouth, outer rostral barbel reaching tip of suborbital spine, maxillary barbel extending almost to vertical line at junction of head and dorsum.

Morphometric data of the type specimen of *P.longibarba* are given in Table 1. Dorsal fin with three unbranched and seven branched rays; pectoral fin with one unbranched and 5–6 branched rays; pelvic fin with one unbranched and four branched rays; anal fin with three unbranched and five branched rays; caudal fin with 12–13 branched rays. Dorsal-fin base short, originating at midpoint of body length, with tip of dorsal fin extending to vertical of anus origin; in male, the first branched pectoral fin ray elongated posteriorly and thicker, with a pointed tip; pelvic-fins origin closer to anal-fin origin than to pectoral-fin base, not reaching anus; anus elongated posteriorly into tube and closer to anal-fin origin; caudal fin emarginate, margins of lobes uneven.

**Table 1.** Morphometric and meristic data of *Protocobitislongibarba* sp. nov.

Characters	Holotype	Paratypes (mean ± SD)
Total length (mm)	53.5	43.7–61.7 (54.8 ± 7.1)
Standard length (mm)	44.0	39.5–51.9 (46.9 ± 5.4)
**Percent of standard length (%)**		
Deepest body depth	8.4	7.6–9.4 (8.8 ± 0.8)
Head width	7.3	7.7–10.3 (8.7 ± 0.9)
Head depth	14.3	9.9–15.1 (13.1 ± 2.2)
Lateral head length	20.4	17.2–21.9 (19.7 ± 1.8)
Prepelvic length	50.9	47.5–52.1 (50.7 ± 1.7)
Preanal length	78.2	71.2–76.7 (74.0 ± 1.9)
Preanus length	71.2	62.9–70.7 (67.9 ± 3.0)
Caudal-peduncle length	17.5	16.8–19.9 (18.1 ± 1.5)
Caudal-peduncle depth	7.2	6.2–9.8 (7.9 ± 1.3)
**Percent of lateral head length (%)**		
Head width	35.8	38.9–55.1 (44.7 ± 6.1)
Head depth	70.2	50.2–80.6 (66.8 ± 11.4)
**Percent of caudal-peduncle length (%)**		
Caudal-peduncle depth	41.1	34.9–58.6 (44.0 ± 9.9)
Fin-ray counts		
Dorsal-fin rays	iii, 7	iii, 7
Pectoral-fin rays	i, 6	i, 5–6
Pelvic-fin rays	i, 4	i, 4
Anal-fin rays	iii, 5	iii, 5
Branched caudal-fin rays	13	12–13

Except for head and abdomen, whole body covered with sparse and minute scales, shallowly embedded in skin surface. Cephalic lateral-line and lateral-line pores absent. Nine to 10 inner gill rakers on first gill arch. Chest and abdominal walls thick and rich in fat. Air bladder absent, no bony bladder. Intestine straight, leading directly to anus. Ribs degenerate, each vertebra with only short and simple parapophysis (Fig. 1I). Vertebrae (from radiograph) 4+42.

#### Coloration.

In life, body generally pale, without pigment, head and all fins transparent, outline of skull visible through skin, barbels exhibit distinct blood vessels (Fig. 1H). Whole body after preservation in formalin pale white, without pigment.

#### Sexual dimorphism.

Male smaller than females, with longer pectoral fin. First branched pectoral fin ray in male thickened and elongated but without the lamina circularis, longest fin ray reaching midpoint between origin of pectoral fin and anus (Fig. 1A–C). First branched pectoral fin ray in females as long as second branched ray (Fig. 1D–F).

#### Etymology.

The specific epithet is a combination of the Latin words *long*- (long) and -*barba* (barbel), indicating its long maxillary barbel, which extends almost to the vertical line at the junction of the head and dorsal body, feminine. We suggest the common Chinese name “Cháng Xū Yuán Huā Qiū (长须原花鳅)” and English name “long-barbal protocobitis”.

#### Distribution and habitat.

**Figure 2.**
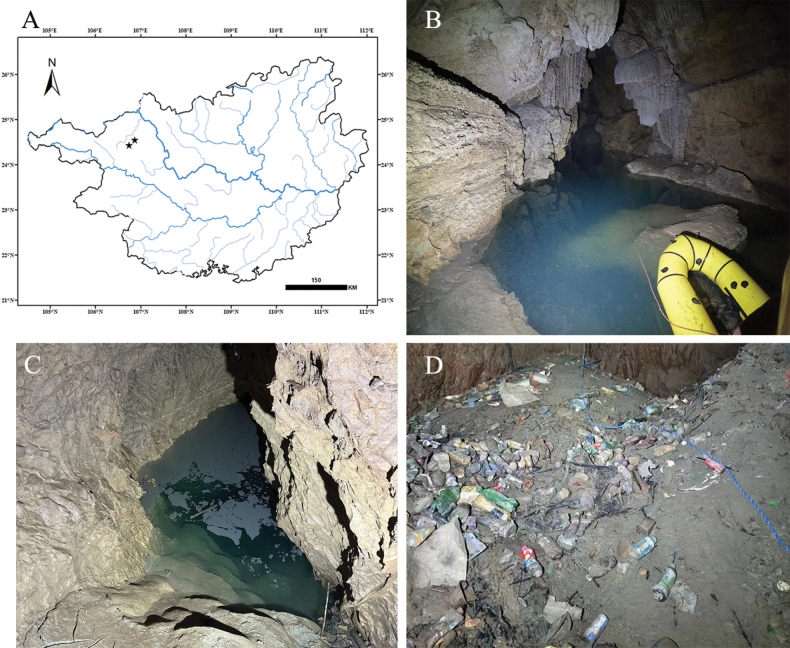
Distribution of *Protocobitislongibarba* sp. nov. and the environment of the cave at the time of collection **A** distribution map of *Protocobitislongibarba* sp. nov. **B** Liangfeng Cave in Fengshan County **C, D** cave in Lingyun County.

The new species is currently known from a cave located in Yangcun Village, Luolou Town, Lingyun County, Baise City and Jinya Township, Fengshan Country, Hechi City (Fig. 2A). In Fengshan County, this species occurs in a pool at the end of cave, which, in the dry season, has an area of approximately 100 m^2^; the water surface is about 15 m from the ground. The water depth is more than 30 m, and the water is clear and unpolluted (Fig. 2B). The pool belongs to the Poxin subterranean river system. Sympatric species include *Sinocyclocheiluslingyunensis* Li, Xiao & Luo, 2000, *Sinocyclocheilusmicrophthalmos* Li, 1989, *Sinocyclocheilusanshuiensis* Gan, Wu, Wei & Yang, 2013, and *Triplophysalingyunensis* Liao,Wang & Luo, 1997. The cave in Lingyun County is approximately 300 m long, inclined downward at a 45° angle. The water pool located at the end of the cave is connected to the Shuiyuan Cave subterranean river system, which are, in turn, connected to a tributary of the Hongshui River. During the dry season, the water level area fluctuates from 5 to 50 m^2^ (Fig. 2C). The cave acts as a conduit for surface water, domestic waste, and mud during the rainy season (Fig. 2D). As such, the primary substrate within the cave is mud. *Protocobitislongibarba* mainly feeds on algae and organic detritus and prefers to burrow into the muddy substrate. Sympatric species include *S.lingyunensis*, *S.microphthalmos*, and *S.anshuiensis*.

#### Genetic comparisons.

Four sequences totaling 1775 bp in from *P.longibarba* were amplified, resulting in the detection of 14 haplotypes. The haplotype matrix consisted of 1,071 invariable sites, 704 variable sites, 387 parsimony informative sites, and 43 singletons.

**Figure 3.**
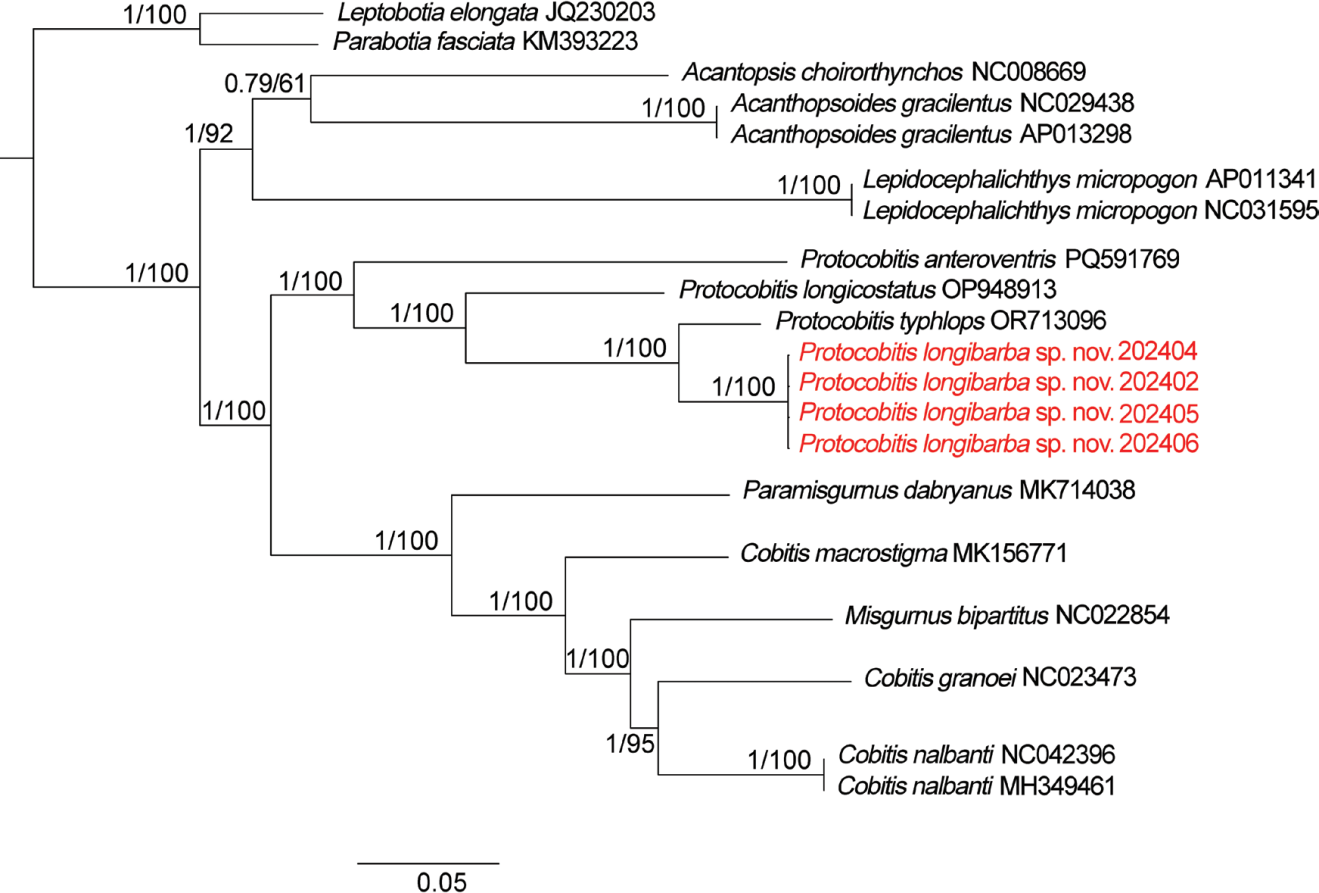
Bayesian phylogram of *Protocobitis* based on concatenated dataset of mitochondrial COI and cyt *b* sequences. The numbers on the nodes represent BPPs from BI and BS from ML.

**Figure 4.**
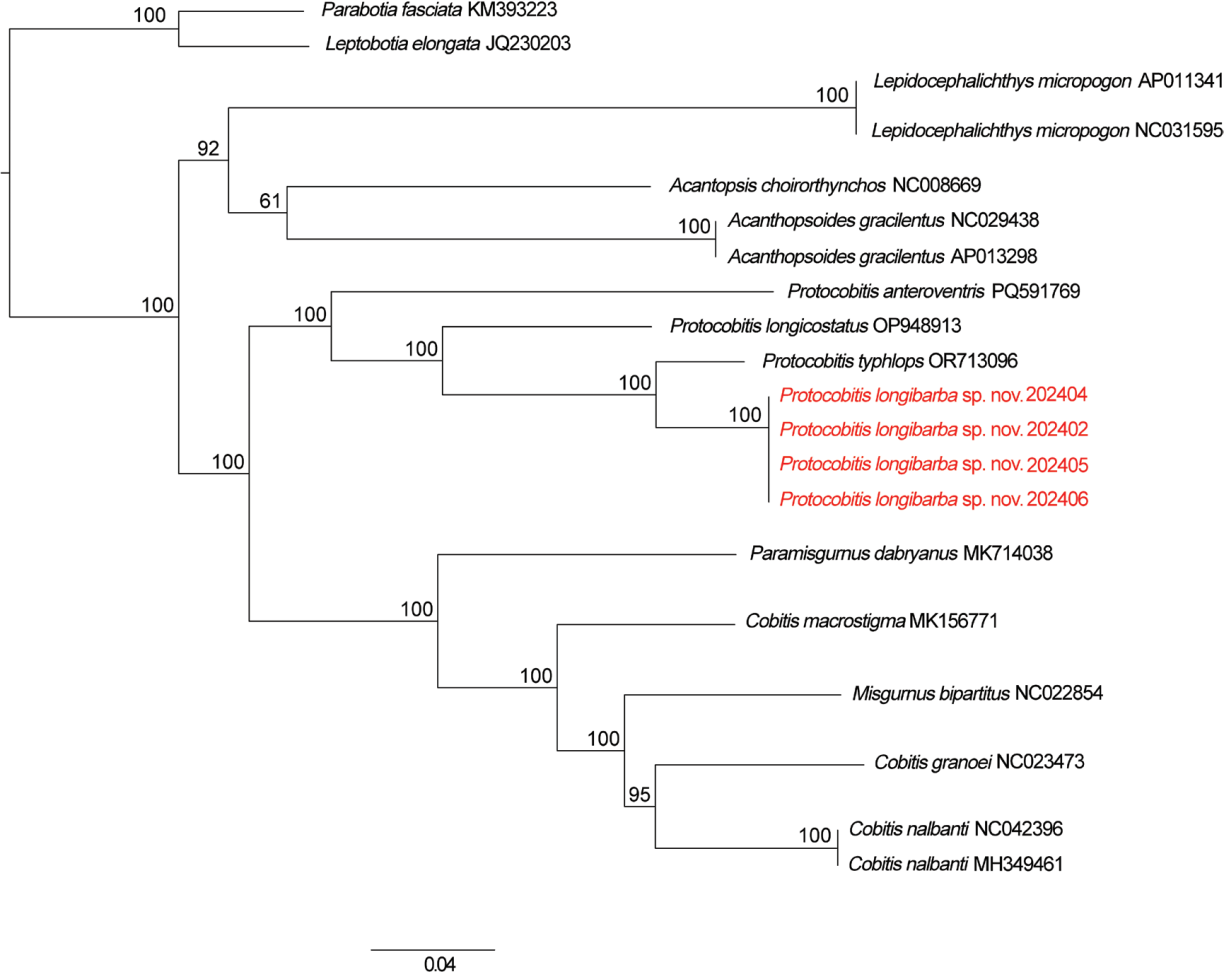
Maximum-likelihood tree of *Protocobitis* based on concatenated dataset of mitochondrial COI and cyt *b* sequences. Numbers near branches indicate bootstrap supports.

The ML and BI phylogenetic trees exhibited congruent topological structures (ML tree see Fig. 4), exhibiting high node support for the monophyly of *P.longibarba* (BPP = 1; BS = 100), which was clustered with the other congeners. *Protocobitis* was sister to the lineage composed of species from *Paramisgurnus* Guichenot, 1872, *Misgurnus* Lacepède, 1803, and *Cobitis* Linnaeus, 1758 (Fig. 3). In the phylogenetic tree, *P.anteroventris* diverged earliest, followed by *P.longicostatus*, while *P.typhlops* was identified as sister group to *P.longibarba*. Additionally, pairwise comparisons of the concatenated dataset of mitochondrial COI and cyt *b* sequences revealed that the uncorrected *p*-distance between species of *Protocobitis* ranged from 6.25% to 16.45%. The minimum uncorrected *p*-distance is between *P.longibarba* and *P.typhlops* (6.25%), and the maximum uncorrected *p*-distance is between *P.longibarba* and *P.anteroventris* (16.45%) (Table 2).

**Table 2.** Uncorrected pairwise distances between species of *Protocobitis* based on concatenated dataset of mitochondrial COI and cyt *b* sequences.

ID	Species	1	2	3
1	*P.typhlops*			
2	*P.longibarba* **sp. nov.**	0.0625		
3	*P.longicostatus*	0.1166	0.1352	
4	*P.anteroventris*	0.1514	0.1645	0.1442

## ﻿Discussion

Both morphological and molecular data support the validity of *Protocobitislongibarba*. The genus *Protocobitis* is typically characterized by the presence of degenerate ribs, with *P.anteroventris*, *P.longicostatus*, and *P.longibarba* showing progressively shorter ribs and *P.typhlops* and *P.polylepis* lacking ribs entirely. Vertebral counts also show variation among the species, with *P.anteroventris* having the highest count (4+57), *P.polylepis* having the lowest count (4+38), and the other three species ranging from 4+42 to 4+43. The differences in vertebral count and rib degeneration indicated adaptations to cave environments. Morphologically, the new species can be distinguished from all other congeners based on a combination of the following characteristics: whole body covered with scales except for head and area between pectoral-fin origin and pelvic-fin origin (vs scaleless in *P.anteroventris*, scales present along midline of body in *P.typhlops*, 5–6 branched pectoral-fin rays (vs seven in *P.anteroventris*, *P.longicostatus*, and *P.polylepis*), and four branched pelvic-fin rays (vs five in other species within the genus). Furthermore, the new species can be distinguished from *P.polylepis* by the absence of pigmentation (vs black pigmentation present), from *P.anteroventris* and *P.polylepis* by caudal peduncle length 17%–20% of SL (vs 25%–28% in *P.anteroventris* and 15%–16% in *P.polylepis*), from *P.longicostatus* and *P.typhlops* by head height 50%–81% of lateral head length (vs 46%–50% in *P.longicostatus* and 52%–61% in *P.typhlops*), and from *P.polylepis* and *P.typhlops* by body height 8%–9% of SL (vs 17%–18% in *P.polylepis* and 9%–14% in *P.typhlops*).

This study provides a comprehensive morphological characters and x-ray scanning and three-dimensional (3D) reconstructions analysis of *P.longibarba*, contributing to the understanding of systematics and adaptations within this genus. The species exhibits distinct morphological characteristics, including rib reduction, which appears to be a consistent and diagnostic feature in *Protocobitis*. This structural adaptation may reflect an evolutionary shift away from reliance on ribs, potentially influencing body stability or flexibility. Additionally, we observed thickening of the chest and abdominal walls and absence of an air bladder, both of which are typical adaptations associated with benthic or bottom-dwelling species. The lack of an air bladder might indicate an ecological specialization, as reduced buoyancy is often advantageous for organisms that inhabit substrates or demonstrate sediment-burrowing behaviors (Longley 1993; Myhre and Klepaker 2009). These morphological traits raise the hypothesis that *P.longibarba* may engage in substrate penetration or mud-burrowing activities, behaviors warranting further ecological investigation to confirm.

Cavefish species exhibit high diversity, making them valuable for studying animal adaptations to extremely dark environments. Cavefish populations are extremely rare and highly sensitive to human disturbances due to their specialized habitats. Minor environmental changes, such as water pollution or extensive human activity, can lead to population extirpation or species extinction. During our field investigation, we observed that the karst cave inhabited by *P.longibarba* functions as a ponor cave in Lingyun City, leading to significant amounts of waste being transported into the cave by the river. This contamination has substantially impacted the habitat, hindering efforts to protect the cave-dwelling organisms. Consequently, establishing effective protection measures is crucial not only for preserving biodiversity but also for safeguarding the natural heritage and potential scientific value represented by cave-dwelling organisms.

## Supplementary Material

XML Treatment for Protocobitis longibarba

## ﻿Additional information

### Author ORCIDs

Zhi-Xian Qin https://orcid.org/0009-0001-7271-813X

Jing-Song Shi https://orcid.org/0000-0001-9168-1734

Li-Na Du https://orcid.org/0000-0002-2246-643X

Jia-Jun Zhou https://orcid.org/0000-0003-1038-1540

### Data availability

All of the data that support the findings of this study are available in the main text.

## References

[B1] Chen YR Yang JX Zhu ZG (1994) A new fish of the genus *Sinocyclocheilus* from Yunnan with comments on its characteristic adapation (Cypriniformes: Cyprinidae). Acta Zootaxonomica Sinica 19(2): 246–253. [In Chinese]

[B2] Fan C Wang M Wang JJ Luo T Zhou JJ Xiao N Zhou J (2024) *Sinocyclocheilusxiejiahuai* (Cypriniformes, Cyprinidae), a new cave fish with extremely small population size from western Guizhou, China. ZooKeys 1214: 119–141. 10.3897/zookeys.1214.127629 39397882 PMC11467493

[B3] Jeffery WR (2001) Cavefish as a Model System in Evolutionary Developmental Biology. Developmental Biology 231(1): 1–12. 10.1006/dbio.2000.0121 11180948

[B4] Jeffery WR (2009) Regressive evolution in Astyanax cavefish. Annual Review of Genetics 43: 25–47. 10.1146/annurev-genet-102108-134216 PMC3594788 19640230

[B5] Kumar S Stecher G Tamura K (2016) MEGA7: molecular evolutionary genetics analysis version 7.0 for bigger datasets. Molecular Biology and Evolution 33(7): 1870–1874. 10.1093/molbev/msw054 27004904 PMC8210823

[B6] Lan JH Gan X Wu TJ Yang J (2013) Cave Fishes of Guangxi. Science Press, Beijing. [In Chinese]

[B7] Liang XF Cao L Zhang CG (2011) Molecular phylogeny of the *Sinocyclocheilus* (Cypriniformes: Cyprinidae) fishes in northwest part of Guangxi, China. Environmental Biology of Fishes 92: 371–379. 10.1007/s10641-011-9847-6

[B8] Librado P Rozas J (2009) DnaSP v5: a software for comprehensive analysis of DNA polymorphism data. Bioinformatics 25: 1451–1452. 10.1093/bioinformatics/btp187 19346325

[B9] Longley LG (1993) Morphological adaptations of the Texas blind catfishes *Trogloglanispattersoni* and *Sataneurystomus* (Siluriformes: Ictaluridae) to their underground environment. Copeia 1993(4): 976–986. 10.2307/1447075

[B10] Miller MA Pfeiffer W Schwartz T (2010) Creating the CIPRES Science Gateway for inference of large phylogenetic trees. In: Proceedings of the Gateway Computing 349 Environments Workshop (GCE), 14 Nov. 2010, New Orleans, LA, 1–8. 10.1109/GCE.2010.5676129

[B11] Myhre F Klepaker T (2009) Body armour and lateral-plate reduction in freshwater three-spined stickleback *Gasterosteusaculeatus*: adaptations to a different buoyancy regime? Journal of Fish Biology 75: 2062–2074. 10.1111/j.1095-8649.2009.02404.x 20738672

[B12] Rambaut A (2009) FigTree. Version 1.4.4. http://tree.bio.ed.ac.uk/software/figtree

[B13] Ronquist F Teslenko M van der Mark P Ayres DL Darling A Höhna S Larget B Liu L Suchard MA Huelsenbeck JP (2012) MrBayes 3.2: efficient Bayesian phylogenetic inference and model choice across a large model space. Systematic Biology 61(3): 539–542. 10.1093/sysbio/sys029 22357727 PMC3329765

[B14] Stamatakis A (2014) RAxML Version 8: a tool for phylogenetic analysis and post-analysis of large phylogenies. Bioinformatics 30(9): 1312–1313. 10.1093/bioinformatics/btu033 24451623 PMC3998144

[B15] Yang JX Chen YR Lan JH (1994) *Protocobitistyphlops*, a new genus and species of cave loach from China (Cypriniformes: Cobitidae). Ichthyological Exploration of Freshwaters 5: 91–96.

[B16] Zhao YH Zhang CG (2006) Cavefishes: concept, diversity and research progress. Bio­diversity Science 5: 451–460. [In Chinese] 10.1360/biodiv.050226

[B17] Zhao Y Zhang C (2009) An Endemic Cavefish Genus *Sinocyclocheilus* in China—Species Diversity, Systematics and Zoogeography (Cypriniformes: Cyprinidae). Science press, Beijing. [In Chinese]

[B18] Zhou JJ Qin ZX Du LN Wu HY (2024) A new species of the blind cave loach genus *Protocobitis* (Cypriniformes: Cobitidae), *Protocobitislongicostatus* sp. nov., from Guangxi, China. Zoological Science 41(2): 210–215. 10.2108/zs230104 38587916

[B19] Zhu Y Lv YJ Yang JX Zhang S (2008) A new blind underground species of the genus *Protocobitis* (Cobitidae) from Guangxi, China. Zoological Science 29: 452–454. 10.3724/SP.J.1141.2008.00452

